# Effect of Neoadjuvant Chemoradiotherapy on Locally Advanced Rectal Mucinous Adenocarcinoma: A Propensity Score-Matched Study

**DOI:** 10.1155/2017/5715219

**Published:** 2017-03-16

**Authors:** Yan-wu Sun, Pan Chi, Hui-ming Lin, Xing-rong Lu, Ying Huang, Zong-bin Xu, Sheng-hui Huang, Xiao-jie Wang

**Affiliations:** Department of Colorectal Surgery, Fujian Medical University Union Hospital, Fuzhou, Fujian 350001, China

## Abstract

*Aims.* To compare the surgical and oncological outcomes of rectal mucinous adenocarcinomas treated with neoadjuvant chemoradiotherapy versus surgery alone.* Methods.* A total of 167 locally advanced rectal mucinous adenocarcinoma patients treated with neoadjuvant chemoradiotherapy and surgery alone between 2008 and 2014 were matched using propensity score; the surgical and oncological outcomes were compared.* Results.* Ninety-six patients were matched. Postoperative morbidity was similar between groups. Sphincter preservation rate was higher in patients receiving neoadjuvant chemoradiotherapy (79.2% versus 60.4%, *P* = 0.045), especially for tumors ≥ 3 cm but ≤5 cm from the anal verge (75.0% versus 44.0%, *P* = 0.036). With a median follow-up of 54.8 months, the 5-year overall survival rate (neoadjuvant chemoradiotherapy versus surgery alone: 79.6% versus 67.1%; *P* = 0.599) and disease-free survival rate (75.6% versus 64.2%; *P* = 0.888) were similar. The 5-year local recurrence rate was lower in patients receiving neoadjuvant chemoradiotherapy (7.7% versus 26.0%, *P* = 0.036), while no difference was observed in distant metastasis. A poor response to chemoradiation was associated with higher local recurrence (*P* = 0.037).* Conclusions.* Compared with surgery alone, neoadjuvant chemoradiotherapy was found to increase the sphincter preservation rate and reduce local recurrence, thus being beneficial for locally advanced rectal mucinous adenocarcinoma patients.

## 1. Introduction

Neoadjuvant chemoradiotherapy (nCRT) followed by curative resection is considered as a standard of care for locally advanced low rectal cancer (LARC). This multimodal treatment has the potential to induce tumor downsizing and tumor downstaging and possibly improve sphincter preservation and local control [1–3]. However, the tumor response varies, ranging from pathological complete response (pCR) to disease progression [4].

Mucinous adenocarcinoma (MAC) is a histological subtype constituting 10–20% of rectal cancers and has been associated with an impaired prognosis compared with nonmucinous adenocarcinoma (non-MAC) [5–7]. Several studies have revealed that rectal MAC exhibits a poor response to chemoradiotherapy and thus worse outcomes compared to non-MAC [8–12]. In contrast, other studies have shown no difference in survival outcomes between the two histological subtypes [9, 13]. Nevertheless, those data were derived from comparisons between rectal MAC and non-MAC treated with nCRT based on nonmatched cohorts. To our knowledge, studies focused on the effect of nCRT on rectal MAC are limited. Currently, no case-matched study has compared the long-term outcomes of rectal MAC treated with nCRT and surgery alone.

Therefore, to examine the benefits of nCRT for the treatment of locally advanced rectal MAC, this propensity score-matched study was designed to compare surgical and oncological outcomes for rectal MAC treated with nCRT and surgery alone. Furthermore, the association between possible survival benefits and the response to nCRT was also evaluated.

## 2. Methods

### 2.1. Patients

This study is a case-matched retrospective study. Between January 2008 and December 2014, 1217 patients with LARC who underwent curative resection were identified from our prospective colorectal cancer database. The inclusion criteria were as follows: (1) clinical T3/T4 or N+ tumors, (2) tumors located within 12 cm from the anal verge, (3) histologically proven adenocarcinoma, and (4) no evidence of distant metastasis. The exclusion criteria were as follows: (1) nonmucinous adenocarcinoma, (2) emergent surgery, palliative resection, or local excision, (3) familial adenomatous polyposis or Lynch syndrome, and (4) previous or concurrent malignancy. This study was approved by the institutional review board. All patients provided written informed consent.

### 2.2. Treatment and Follow-Up

Abdominopelvic magnetic resonance imaging (MRI) and/or transrectal ultrasonography were used to evaluate tumor resectability. Generally, nCRT was chosen if the tumor required downsizing for clear surgical margins or sphincter preservation. Nevertheless, the final decision whether to perform nCRT or surgery alone was made by the patients based on the current stage of their disease and after understanding the risks and benefits of each group and without the influence of the surgeons. The preoperative long-course radiotherapy protocol consisted of 50.4 Gy delivered in fractions of 1.8 Gy 5 times per week for 5 consecutive weeks followed by a 5.4 Gy boost. Preoperative chemotherapy was initiated on the first day of radiotherapy and included two different regimens: FOLFOX (5-FU/folinic acid/oxaliplatin) and CapeOX (capecitabine and oxaliplatin).

Surgery was performed 6 to 8 weeks after completion of radiation. Surgical techniques for rectal cancer, such as total mesorectal excision (TME) and high ligation of the inferior mesenteric artery, were standardized at our institution. The surgical procedures used were as follows: low anterior resection (LAR), abdominoperineal resection (APR), and Hartmann's procedure. A diverting ileostomy was performed at the surgeon's discretion based on several factors, including the general health of the patient, the distance of the anastomosis from the anal verge, and the use of nCRT. Starting at approximately 3 to 4 weeks after surgery, all patients received postoperative adjuvant chemotherapy for 6 months, including the two different regimens (FOLFOX and CapeOX).

Postoperative follow-up was conducted every 3 months for the first 3 years, then every 6 months for the next 2 years, and annually thereafter. At each visit, a physical examination, CEA, chest X-ray, or CT, and abdominopelvic MRI or CT scans were performed. A colonoscopy was performed annually after surgery. Positron emission tomography (PET) was performed when needed. Patient follow-up lasted until death or until the cut-off date of March 30, 2016.

### 2.3. Measurements and Definitions

Pathological specimens were examined by at least two experienced pathologists. Mucinous adenocarcinoma was diagnosed when more than 50% of the tumor exhibited mucinous features, consisting of nests of adenocarcinoma cells immersed in mucin pools [14]. To exclude a mucinous phenotype induced by radiotherapy, tumors with acellular mucin pools were also evaluated on pretreatment MRI scans (when available) by a radiologist.

Tumor regression was graded according to the Rectal Cancer Regression Grade (RCRG) method by Wheeler et al. [15]. A pCR was defined as the absence of tumor cells in the pathologic specimen, either at the primary site or in the lymph nodes. Postoperative complications were graded according to the Clavien-Dindo classification [16]. Perioperative mortality was defined as any death either within 30 days of surgery or during the hospitalization period. Locoregional recurrence was defined as any tumor relapse within the pelvis, perineum, or anastomosis, whereas a distant metastasis was defined as any other recurrence.

### 2.4. Statistical Analysis

Propensity score analysis was performed using R project for Statistical Computing, Version 2.12.1 (R Development Core Team, Vienna, Austria) along with SPSS Essentials for R 20. Logistic regression was used to estimate the propensity scores for each group. Covariates in the model used to determine propensity scores included age, gender, tumor location, tumor diameter, clinical T stage, clinical N stage, and pretreatment CEA level.

Statistical analyses were performed using SPSS version 20.0 (SPSS Inc., Chicago). Categorical variables were expressed as numbers with percentages and compared using a Chi-square test or Fisher's exact test when appropriate. Normally distributed data were described by means ± standard deviations and analysed using Student's *t*-tests. The Kaplan-Meier method was used to assess survival outcomes. The log-rank test was used to compare survival between groups. Statistical significance was defined as *P* < 0.05.

## 3. Results

### 3.1. Patient Characteristics

A total of 167 locally advanced rectal MAC patients were considered for our analysis. After using 1 : 1 propensity score matching, 48 patients treated with nCRT and 48 patients treated with surgery alone were matched in our final analysis. No significant differences were observed between the two groups in terms of age, gender, ASA grade, body mass index, distance from the anal verge, tumor gross type, clinical T stage, clinical N stage, and pretreatment CEA level (Table 1).

### 3.2. Surgical Outcomes

There were no statistical differences between the two groups concerning operative time, estimated blood loss, conversion rate, and surgical approach (Table 2). Three patients in the nCRT group were converted to an open procedure (due to difficulties in pelvic exposure in two cases and severe adhesion in one case). One patient in the surgery-alone group was converted due to severe adhesion. The surgical procedure differed between the groups. A total of 79.2% of patients in the nCRT group underwent LAR compared to 60.4% in the surgery-alone group (*P* = 0.009), while APR was performed less often in the nCRT group (12.5% versus 37.5%).

The sphincter preservation rate was higher in the nCRT group (79.2% versus 60.4%; *P* = 0.045), as was the rate of temporary diverting ileostomy (52.1% versus 22.9%; *P* = 0.003). A subgroup analysis showed that the proportion of sphincter-sparing surgery among patients with tumors ≥ 3 cm but ≤5 cm from the anal verge was significantly higher in the nCRT group (75.0% versus 44.0%, *P* = 0.036). The sphincter preservation rate among patients with tumors > 5 cm from the anal verge was similar between the two groups (85.2% versus 94%; *P* = 0.628). No patient in either group with a tumor < 3 cm from the anal verge underwent sphincter-sparing surgery.

### 3.3. Pathological Results

Compared to surgery alone, nCRT decreased the number of lymph nodes retrieved and the number of positive lymph nodes (*P* = 0.001 and *P* = 0.046). A positive circumferential resection margin (CRM) was more commonly observed in the surgery-alone group (10.4% versus 2.1%), but without significant difference (*P* = 0.204). A positive CRM was observed in both groups for pT3 and pT4 tumors only.

Tumor downstaging was observed in 21 patients (43.8%) in the nCRT group, while nodal downstaging was seen in 24 patients (50%). Two patients (4.1%) achieved a pCR. A good (RCRG1) or partial (RCRG2) response was observed in 15 (31.3%) and 20 (41.6%) patients, respectively, while 13 patients (27.1%) did not respond to chemoradiotherapy (RCRG3).

### 3.4. Surgical Complications

Postoperative morbidity was similar in patients treated with nCRT and surgery alone (31.2% versus 29.2%, *P* = 0.824). The pattern of morbidity differed between the two groups. Anastomotic leakage was more commonly observed in the nCRT group (8.3% versus 4.2%, *P* = 0.674); however, none of the patients required surgery (ileostomy). The incidence of surgical site-related infection presented a trend that favoured the surgery-alone group (10.4% versus 4.2%), but it did not reach significance (*P* = 0.432). Eight patients (16.7%) in the nCRT group were found to have major complications (Clavien-Dindo III/IV) compared to 5 patients (10.4%) in the surgery-alone group, but without significant difference. No significant difference was found for the length of the postoperative hospital stay. No reoperation was performed due to postoperative complications. One patient in the surgery-alone group died of multiple organ failure 5 days after surgery (Table 3).

### 3.5. Oncological Outcomes

The mean follow-up period was 54.8 ± 31.1 months. The 5-year overall survival (OS) for all patients was 72.5%, with 11 deaths in the nCRT group and 17 deaths in the surgery-alone group. The 5-year disease-free survival (DFS) for all patients was 68.3%, with 12 recurrences in the nCRT arm and 16 events in the surgery-alone arm. No significant differences were found in the pattern of recurrence, time to recurrence, and need for additional therapy between the groups (Table 4).

**Figure 1:**
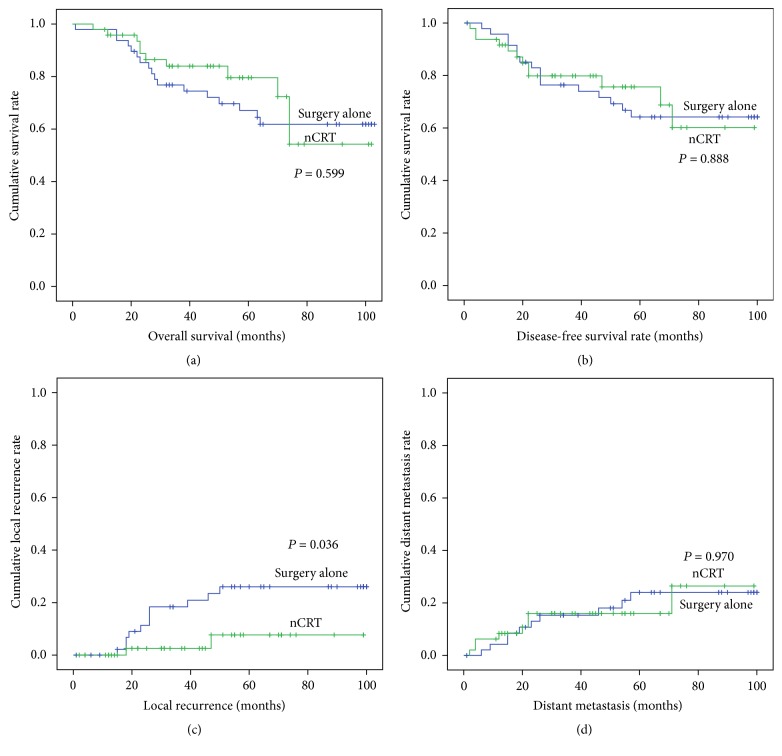
(a) Overall survival, (b) disease-free survival, (c) cumulative local recurrence, and (d) cumulative distant metastasis rate between nCRT group and surgery-alone group. nCRT: neoadjuvant chemoradiotherapy.

**Figure 2:**
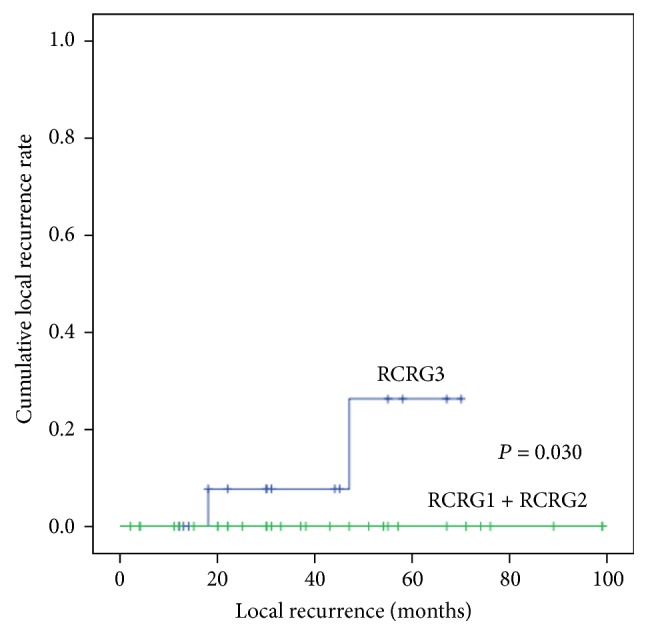
Cumulative local recurrence rate in nCRT group between RCRG1 + RCRG2 and RCRG3. RCRG: rectal cancer regression grade; nCRT: neoadjuvant chemoradiotherapy.

The 5-year OS rate (nCRT versus surgery alone: 79.6% versus 67.1%; *P* = 0.599) and DFS rate (75.6% versus 64.2%; *P* = 0.888) were similar between the two groups (Figure 1). The 5-year cumulative local recurrence rate was 7.7% in the nCRT group, which was significantly lower than the rate in the surgery-alone group (26.0%; *P* = 0.036). No difference was observed in the 5-year distant metastasis rates between the groups (nCRT versus surgery alone: 16.0% versus 24.0%; *P* = 0.970). Moreover, a significant correlation was found between tumor response and local recurrence, and the 5-year local recurrence was higher in nonresponders (RCRG3) than in responders (RCRG1 + RCRG2) (30% versus 0%, *P* = 0.037) (Figure 2).

## 4. Discussion

Few studies have focused on the effect of nCRT on locally advanced rectal MAC. This case-matched study demonstrated that nCRT for the treatment of locally advanced rectal MAC provided an acceptable tumor response, together with a higher sphincter preservation rate and a lower local recurrence, when compared with surgery alone.

It has been reported that surgeons might encounter increased intraoperative difficulty and surgical morbidity in LARC patients after nCRT [1, 17], while others have shown that nCRT does not increase surgical morbidity or mortality [18, 19]. In this series, we did not encounter greater intraoperative difficulty and more postoperative complications in rectal MAC patients receiving nCRT, which might be attributed to the high volume and extensive surgical experience of our specialized center. It has been noted that nCRT may result in significant morbidity, such as anastomotic leakage and wound infection [1, 20, 21]. Although a diverting ileostomy may decrease morbidity associated with anastomotic leakage, it might increase ileostomy-related complications, including small bowel obstruction, stoma necrosis, prolapse, or retraction [22, 23]. However, this is balanced against the risk of anastomosis-related morbidity at rectal resection. Postoperative morbidity was similar in the two groups, and we did not find a greater incidence of anastomotic leakage in patients undergoing nCRT, indicating that nCRT was safe and feasible for rectal MAC.

The overall rate of sphincter preservation in our study (72%) was similar to that reported in previous phase III studies (60%–75%) [24, 25]. We noted a higher sphincter preservation rate in patients treated with nCRT, especially in those with tumors ≥ 3 cm but ≤5 cm from the anal verge. Whether to perform a sphincter-preserving surgery or not remains a complex assessment, which must take into account oncologic adequacy, technical considerations, anal sphincter function, and patient preference. In our series, we used several modalities including digital rectal examination, endorectal ultrasonography (ERUS), pelvic magnetic resonance imaging (MRI), and rectoscopic/colonoscopic examination to evaluate tumor response following nCRT. Reevaluation using intraoperative proctoscopy examination by a surgeon was routinely performed for the purpose of locating the tumor and identifying tumor shrinkage or downstaging. Shrinkage and downstaging of the rectal tumor with nCRT lengthened the distance between the anorectal ring and the lower edge of the tumor, ensured a safe anastomosis, and facilitated sphincter-preserving surgery.

In the neoadjuvant setting, we used the addition of oxaliplatin to a preoperative fluoropyrimidine-based radiotherapy regimen. We showed that tumor and nodal downstaging were 43.8% and 50%, respectively, compared to 30% to 60% for rectal non-MAC in previous studies [5, 8]. Good and partial responses were observed in 31.3% and 41.6% of patients, respectively. Our results indicated that this therapeutic paradigm could provide an acceptable tumor response. Preliminary results from 5 large RCTs have shown that oxaliplatin increases toxicity without conferring a survival benefit in the neoadjuvant treatment of LARC [24–28]. Unfortunately, little is known about the mechanisms responsible for the potential radiosensitivity of oxaliplatin for chemoradiotherapy of rectal MAC. The good tumor response of rectal MAC in the present study conflicts with other reports in the literature [8–10], and it remains unclear whether this response is attributable to the addition of oxaliplatin or to radiosensitivity.

Most studies have reported that the number of lymph nodes retrieved was reduced after nCRT [29, 30], which was also observed in the present study (nCRT versus surgery alone, 13.9 ± 7.7 versus 20.0 ± 9.1, *P* = 0.001). The lymph nodes harvest reflects surgical radicality and adequate pathologic examination [31]. Nevertheless, the average lymph nodes harvest in both groups was well in excess of the current recommendations for 12 lymph nodes [32].

Conflicting results have been reported regarding the tumor response to nCRT between rectal MAC and non-MAC [8–12], as shown in Table 5. The lack of consensus may be attributable to heavy bias in patient selection and the disparity of the defining criteria for MAC. The largest analysis of factors predicting the tumor response to nCRT found that mucinous histology was not predictive of the response to chemoradiotherapy [33]. Little is known about the mechanisms responsible for the poor chemoradiotherapy response of rectal MAC, but it is likely that the different molecular and genetic features (compared to non-MAC), including a higher rate of mutated K-ras, microsatellite instability (MSI), loss of heterozygosity (LOH), and abnormal expression of E-cadherin, are linked to decreased chemoradiosensitivity [34–36]. Unfortunately, these molecular signatures were unknown for patients in our study so that these analyses could not be performed.

Data concerning the prognosis of MAC remain controversial. Some studies have found a worse prognosis associated with this lesion [37, 38], whereas others have not observed a prognostic significance of mucinous histology in colorectal cancer [39, 40]. Several studies have reported a poor response of rectal MAC to nCRT, leading to a poor prognosis when compared with rectal non-MAC [10, 12], whereas others reported no difference [9, 13]. Our study revealed no significant difference in OS and DFS in rectal MAC between the two groups. Nevertheless, the local recurrence rate was significantly lower in the nCRT group than in the surgery-alone group. It has been noted that the degree of tumor response to preoperative chemoradiotherapy is correlated with local recurrence and possibly OS [41]. This study also demonstrated that a poor response to chemoradiation was associated with a higher 5-year local recurrence rate (*P* = 0.037). These findings provide indirect evidence for the effectiveness of nCRT for rectal MAC.

To our knowledge, this study is the first propensity score-matched study demonstrating the benefit of nCRT in treatment of rectal MAC. However, this study has several limitations. This study was subject to inherent selection biases due to the retrospective nature, although we minimized this bias by using propensity score matching. The small sample size, likely due to the low incidence of rectal MAC, limited the statistical power of our study. Additionally, the diagnosis of MAC was made by postoperative pathological examination and preoperative endoscopic biopsy. Preoperative diagnosis based on pretreatment MRI scans may be useful to exclude radiation-induced MAC. Nevertheless, to exclude the mucinous phenotype induced by radiotherapy, tumors with acellular mucin pools were also evaluated on pretreatment MRI scans (when available). Given these limitations, we believe that this propensity-scored, case-matched study could serve as helpful background research for future RCTs investigating nCRT in rectal MAC patients.

## 5. Conclusion

This study suggests that, in case-matched cohorts of local advanced rectal MAC patients, nCRT combined with curative resection can be beneficial in terms of higher sphincter preservation rates and lower local recurrence rates. Neoadjuvant chemoradiotherapy should be recommended for rectal MAC. However, large prospective trials are necessary to determine the efficacy of nCRT for rectal MAC therapy.

## Figures and Tables

**Table 1:** Patient characteristics in unmatched and propensity-matched rectal mucinous adenocarcinomas.

Characteristics	Unmatched patients			Propensity-matched patients		
	nCRT (*n* = 61)	Surgery alone (*n* = 103)	*P* value	nCRT (*n* = 48)	Surgery alone (*n* = 48)	*P* value
Gender			0.094			0.682
Male	39 (63.9)	52 (50.5)		27 (56.2)	25 (52.1)	
Female	22 (36.1)	51 (49.5)		21 (43.8)	23 (47.9)	
Age (years)	54.9 ± 13.8	54.7 ± 14.7	0.918	55.9 ± 14.2	54.1 ± 14.7	0.540
ASA score			0.418			0.787
1	33 (54.1)	52 (50.5)		24 (50.0)	26 (54.2)	
2	21 (34.4)	44 (34.4)		18 (37.5)	18 (37.5)	
3	7 (11.5)	7 (6.8)		6 (12.5)	4 (8.3)	
BMI (kg/m^2^)	23.2 ± 4.8	22.3 ± 2.8	0.122	22.9 ± 3.1	22.6 ± 2.7	0.626
Distance from the anal verge (cm)	5.7 ± 1.7	6.9 ± 2.8	**0.005**	5.8 ± 1.8	5.5 ± 2.5	0.455
Tumor diameter (cm)	3.4 ± 1.0	4.2 ± 1.5	**<0.001**	3.5 ± 1.1	3.7 ± 1.0	0.461
Gross type			0.156			0.565
Expanding	27 (26.2)	13 (21.3)		12 (25.0)	12 (25.0)	
Ulcering	72 (69.9)	41 (67.2)		30 (62.5)	33 (68.8)	
Infiltrating	4 (3.9)	7 (11.5)		6 (12.5)	3 (6.2)	
Clinical T stage			**0.047**			0.637
T3	15 (24.6)	41 (39.8)		13 (27.1)	11 (22.9)	
T4	46 (75.4)	60.2 (60.2)		25 (72.9)	37 (77.1)	
Clinical N stage			0.782			0.695
N0	8 (13.1)	12 (11.7)		4 (8.3)	3 (6.2)	
N+	53 (86.9)	91 (88.3)		44 (91.7)	45 (93.8)	
Pretreatment CEA level			0.256			0.404
Normal (<5 ng/mL)	33 (54.1)	65 (63.1)		27 (56.2)	31 (64.6)	
Elevated (≥5 ng/mL)	28 (45.9)	38 (36.9)		21 (43.8)	17 (35.4)	

nCRT: neoadjuvant chemoradiotherapy; ASA: American Society of Anesthesiologists; BMI: body mass index.

Data are expressed as *n* (%) or as median ± standard deviation, where appropriate. Row percents are presented. Significant differences are in bold.

**Table 2:** Surgical and pathological outcomes between nCRT and surgery-alone groups.

Characteristics	nCRT (*n* = 48)	Surgery alone (*n* = 48)	*P* value
Operative time (min)	221.7 ± 62.1	225.6 ± 65.8	0.765
Estimated blood loss (mL)	152.4 ± 189.9	169.7 ± 160.2	0.631
Conversion rate	3 (6.2)	1 (2.1)	0.617^a^
Surgical approach			0.837
Laparoscopy	22 (45.8)	21 (43.8)	
Open	26 (54.2)	27 (56.2)	
Surgical procedure			**0.009**
LAR	38 (79.2)	29 (60.4)	
APR	6 (12.5)	18 (37.5)	
Hartmann's procedure	4 (8.3)	1 (2.1)	
Sphincter preservation	38 (79.2)	29 (60.4)	**0.045**
Temporary diverting ileostomy	25 (52.1)	11 (22.9)	**0.003**
Length of resection margin (cm)			
Proximal	14.6 ± 2.0	14.9 ± 0.8	0.461
Distal	3.0 ± 1.1	3.2 ± 1.4	0.612
Number of lymph nodes retrieved	13.9 ± 7.7	20.0 ± 9.1	**0.001**
Number of positive lymph nodes	2.9 ± 7.1	5.9 ± 7.4	**0.046**
Resection margin involvement			
Distal	0	1 (2.1)	NA
CRM (≤1.0 mm)	1 (2.1)	5 (10.4)	0.204^a^
Neural or lymphovascular invasion	4 (8.3)	6 (12.5)	0.740^a^
pTNM stage			< 0.001
0	2 (4.2)	0	
I	7 (14.6)	0	
II	15 (31.2)	3 (6.2)	
III	24 (50.0)	45 (93.8)	
Rectal cancer regression grade			
1	15 (31.3)	NA	NA
2	20 (41.6)	NA	NA
3	13 (27.1)	NA	NA
Tumor downstaging	21 (43.8)	NA	NA
Nodal downstaging	24 (50.0)	NA	NA
Pathological complete response	2 (4.1)	NA	NA

^a^Fisher's exact test.

Data are expressed as *n* (%) or as median ± standard deviation, where appropriate. Row percents are presented. Significant differences are in bold.

nCRT: neoadjuvant chemoradiotherapy; LAR: low anterior resection; APR: abdominoperineal resection; NA: not applicable; CRM: circumferential resection margin.

**Table 3:** Postoperative morbidity between nCRT and surgery-alone groups.

Characteristics	nCRT (*n* = 48)	Surgery alone (*n* = 48)	*P* value
Postoperative hospital stay	10.8 ± 6.0	11.8 ± 5.6	0.410
Postoperative mortality	0	1 (2.1)	NA
Overall morbidity	15 (31.2)	14 (29.2)	0.824
Postoperative complications^a^			
Anastomotic leakage	4 (8.3)	2 (4.2)	0.674
Abdominal bleeding	1 (2.1)	0	NA
Ileus	5 (10.4)	2 (4.2)	0.432
Acute urinary retention	0	1 (2.1)	NA
Wound infection	2 (4.2)	5 (10.4)	0.432
Pulmonary infection	6 (12.5)	4 (8.3)	0.504
Sepsis	2 (4.2)	0	NA
Multiple organ failure	0	1 (2.1)	NA
Reoperation	0	0	NA
Grade of morbidity			
Minor	7 (14.6)	9 (18.8)	0.584
Major	8 (16.7)	5 (10.4)	0.371

^a^Patients may have experienced more than one complication.

nCRT: neoadjuvant chemoradiotherapy; NA: not applicable.

**Table 4:** Recurrence patterns between nCRT and surgery-alone groups.

Outcomes	nCRT (*n* = 48)	Surgery alone (*n* = 48)	*P* value
Number of patients with recurrence	12 (25%)	16 (33.3%)	0.369
Recurrence site^a^			0.070
Locoregional	2	11	
Liver	5	4	
Lung	5	3	
Peritoneal	0	2	
Bone	1	2	
Brain	0	2	
Time to recurrence (months)	25.3 ± 23.6	27.9 ± 16.2	0.732
Additional treatment for recurrence			0.921
Resection	4	6	
Chemotherapy	5	7	
Best support care	3	3	

^a^Some patients had more than one recurrence site.

nCRT: neoadjuvant chemoradiotherapy.

**Table 5:** Previously reported outcomes between mucinous and nonmucinous rectal cancer treated with neoadjuvant chemoradiotherapy.

Year	Author	Patients	Response to chemoradiotherapy	*P* value	Long-term survival			
					5-year OS (%)	*P* value	5-year DFS (%)	*P* value
2006	Sengul et al. [8]	Mucinous, 16	TRG1, 1 (6%); TRG2, 2 (12.5%); TRG3, 3 (19%); TRG4, 6 (37.5%); TRG5, 4 (25%)	0.002 (TRG)	NA	NA	NA	NA
			T downstaging, 0; N downstaging, 18%	0.009 (T downstaging)	NA		NA	
		Nonmucinous, 46	TRG1, 6 (13%); TRG2, 23 (50%); TRG3, 6 (13%); TRG4, 11 (24%); TRG5, 0					
			T downstaging 25%, N downstaging 76%	0.006 (N downstaging)				

2007	Grillo-Ruggieri et al. [9]	Mucinous, 25	T downstaging 52%, N downstaging 64%	<0.05^*∗*^	89	NS	87.3	NS
		Nonmucinous, 111	T downstaging 74.4%, N downstaging 85.5%		83.9		67.6	

2011	Shin et al. [10]	Mucinous, 23	T downstaging 54.9%, N downstaging 52.2%	0.03 (T downstaging)	64.8	0.049	58.7	0.045
		Nonmucinous, 345	T downstaging 30.4%, N downstaging 53.3%	NS (N downstaging)	79.8		69.2	

2012	Oberholzer et al. [11]	Mucinous, 21	T downstaging 18.8%, N downstaging 27.2%	0.012 (T downstaging)	NA	NA	NA	NA
		Nonmucinous, 67	T downstaging 55.2%, N downstaging 80.7%	0.007 (N downstaging)	NA		NA	

2014	Yu et al. [12]	Mucinous, 60	T downstaging 23%	0.01	69	0.04	48	0.006
		Nonmucinous, 270	T downstaging 40%		79		71	

2015	Hugen et al. [13]	Mucinous, 58	T downstaging 55.2%	0.039	64.3	0.459	NA	NA
		Nonmucinous, 482	T downstaging 68.7%		70.6		NA	

TRG: tumor regression grade; T: depth of tumor invasion; N: lymph node status; NA: not available; NS: not significant.
